# Mitochondrial DNA D-Loop Polymorphisms among the Galla Goats Reveals Multiple Maternal Origins with Implication on the Functional Diversity of the HSP70 Gene

**DOI:** 10.1155/2024/5564596

**Published:** 2024-02-05

**Authors:** Ednah M. Masila, Stephen O. Ogada, Irene N. Ogali, Grace M. Kennedy, Eric K. Too, Cecily S. Ommeh

**Affiliations:** ^1^Institute for Biotechnology Research, Jomo Kenyatta University of Agriculture and Technology, P.O. Box 62000-00200, Juja, Kenya; ^2^Veterinary Science Research Institute (VSRI), Kenya Agricultural Livestock and Research Organization (KALRO), P.O. Box 32-00902, Nairobi, Kenya

## Abstract

Despite much attention given to the history of goat evolution in Kenya, information on the origin, demographic history, dispersal route, and genetic diversity of Galla goats remains unclear. Here, we examined the genetic background, diversity, demographic history, and population genetic variation of Galla goats using mtDNA D-loop and HSP70 single-nucleotide polymorphism markers. The results revealed 90 segregating sites and 68 haplotypes in a 600-bp mtDNA D-loop sequence. The overall mean mitochondrial haplotype diversity was 0.993. The haplotype diversities ranged between 0.8939 ± 0.0777 and 1.0000 ± 0.0221 in all populations supporting high genetic diversity. Mitochondrial phylogenetic analysis revealed three Galla goat haplogroups (A, G, and D), supporting multiple maternal ancestries, of which haplogroup A was the most predominant. Analysis of molecular variance (AMOVA) showed considerable variation within populations at 94.39%, evidence of high genetic diversity. Bimodal mismatch distribution patterns were observed while most populations recorded negative results for Tajima and Fu's Fs neutrality tests supporting population expansion. Genetic variation among populations was also confirmed using HSP70 gene fragment sequences, where six polymorphic sites which defined 21 haplotypes were discovered. Analysis of molecular variance revealed a significant FST index value of 0.134 and a high FIS index value of 0.746, an indication of inbreeding. This information will pave the way for conservation strategies and informed breeding to improve Galla or other goat breeds for climate-smart agriculture.

## 1. Introduction

Domestic goats (*Capra hircus*) are believed to have been domesticated between the ninth and eighth millennia BC in the Zagros Mountains in Iran and the high Euphrates Valley and later dispersed worldwide [1]. Previous genetic studies have grouped goats into different haplogroups based on their origin/ancestor. Most haplogroups have been found in Asia, Europe, and Africa; however, haplogroup A is the most dominant consisting of more than 90% of goats worldwide [2, 3]. Currently, the Kenyan goat population is estimated to be over 27 million and is mainly classified as indigenous or exotic [4]. Due to their resilience and adaptation capability to various climatic conditions, these goats are primarily distributed in almost every agroclimatic zones of Kenya [5]. For our agricultural production systems to adapt and flourish, a diverse portfolio of animal genetic resources is essential. The necessity to maintain this adaptability is highlighted by climate change and the introduction of new and virulent animal diseases. Therefore, genetic diversity studies promote the production of livestock which is crucial for achieving the sustainable development goals and ensuring the security of food and livelihoods [5].

Goat keeping is essential for the rural communities' daily livelihood, especially in arid and semiarid areas of Kenya where crop farming activities are not feasible. They are mainly kept as a source of income, food, manure, skin, and social-cultural activities such as dowry payments and sacrificial offerings [6]. Considering the growing land shortage due to the increased human population growth rate, there is a need to increase food production [7]. However, despite their wide distribution, indigenous goats are postulated to have low productivity. Attempts to enhance their productivity have mainly concentrated on crossbreeding with exotic germplasms, which is not sustainable. Selective breeding using the adapted indigenous breeds would have a feasible impact on the productivity of the animals [8].

As a result of climate change, prolonged drought periods with increased high temperatures have worsened in arid and semiarid lands (ASALs). This calls for more resilient and adapted livestock breeds to ensure productivity. Galla goats are indigenous to Northern Kenya regions which are dry and experience high temperatures. Consequently, Galla goats already show heat tolerance attributes. Despite the harsh conditions, Galla goats gain weight up to 70 kgs for bucks and 55 kgs for does [9]. This gives them an eminence in meat production and also good sources of milk. Like other organisms, goats possess conserved proteins called heat shock proteins (HSPs), produced when subjected to high ambient temperature. The proteins protect body cells against detrimental outcomes of heat stress hence facilitating the organism's survival. Single-nucleotide polymorphisms (SNPs) in the HSP70 gene have been shown to have a role in thermotolerance [10]. A study by Nikbin et al. [11] reported two heat shock protein 70 SNPs associated with thermoregulation for good-quality sperms in Boer goats.

There is scanty information on Kenyan Galla goats' genetic resources from previous studies. Information from such studies is crucial in the proper utilization of genetic resources and in designing and implementing conservation and improvement programs for indigenous goats. Our study, therefore, aimed to characterize the diversity present among the Galla goats, their genetic background, and their demographic history.

## 2. Materials and Methods

### 2.1. Permits of Compliance

This study received ethical clearance from the Institutional Animal Care and Use Committee (IACUC) in the Veterinary Science Research Institute (VSRI), Kenya Agricultural and Livestock Research Organization (KALRO), under permit number KALRO-VSRI/IACUC022/04062021, to sample goats and permission to conduct the study from the Directorate of Veterinary Services in the Ministry of Agriculture, Livestock, Fisheries, and Cooperatives (MoALFC). In addition, clearance was also sought from the three county governments (Isiolo, Garissa, and Tana River) and prior informed consent from the farmers.

### 2.2. Sample Collection and Deoxyribonucleic Acid (DNA) Extraction

**Figure 1:**
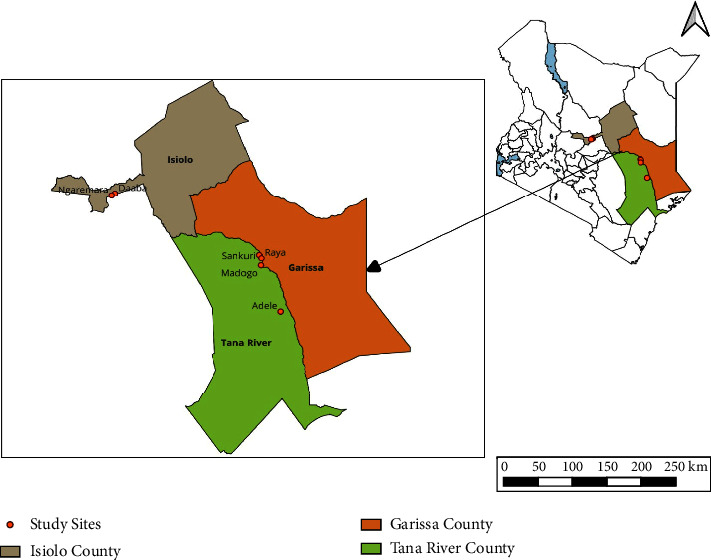
Study area map. The map shows part of Kenya ASAL areas where Galla goats are local inhabitants.

**Figure 2:**
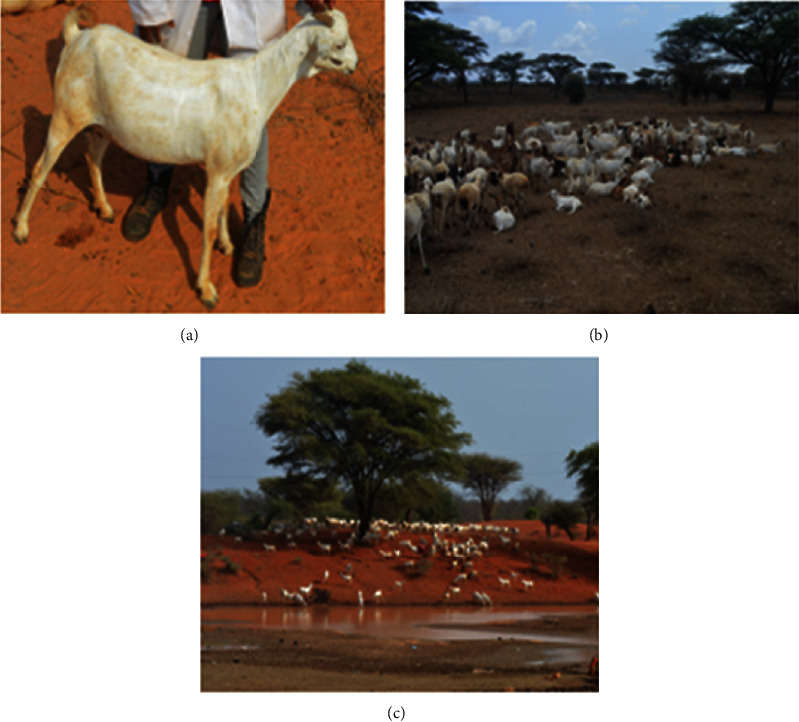
Galla goats and their surrounding environment: (a) typical Galla goat, (b) production environment of the Galla goats in the ASALs, and (c) animal watering point.

96 unrelated Galla goat blood samples were collected from Isiolo (*n* = 32), Garissa (*n* = 32), and Tana River (*n* = 32) counties (Figure 1). Each population included 16 females and 16 males (Table 1). The counties were chosen because goat production has the most significant impact on the livelihoods of their inhabitants. The choice was also based on the ASAL nature of the production environment in the counties (Figure 2).

Total genomic DNA was isolated from whole blood using the Quick-DNA Miniprep kit (Zymo Research) according to the manufacturer's protocol. Gel electrophoresis was performed to confirm the presence of DNA. The concentration and purity of extracted DNA were determined by using the NanoDrop 1000 spectrophotometer [12].

### 2.3. Polymerase Chain Reaction (PCR) Amplification and Sequencing

The primer pair 600bp-F 5′ CATCCATATAACGCGGACAT-3′ and 8-R 5′- GTGTGAGCATGGGCTGATTA -3′ flanking the mtDNA's first hypervariable region (HVR-1) were designed for amplification based on Okpeku et al. [13]. The HSP70 gene was amplified using the following primer sequences: forward 5′ ATGGCGAAAAACATGGCTATC and reverse 5′ CTAATCCACCTCCTCAAT as previously described by Gade et al. [14]. The complete HSP70.1 gene (1926 bp) was amplified. PCR amplification was performed in a total volume of 50 *μ*l containing 10 *μ*M of each primer and 5 *μ*l of DNA template. Amplification was carried out in a Veriti™ 96-well thermal cycler. The PCR amplicons were assessed on a 2% agarose gel stained with ethidium bromide and visualized under ultraviolet light. The amplicons were sent for purification and sequencing at Macrogen, Netherlands (Europe). Ninety-six sequences were sequenced for both mtDNA and HSP70 genes.

## 3. Data Analysis

### 3.1. Mitochondrial DNA (MtDNA) and HSP70 Sequence Editing and Diversity Indices

Forward and reverse sequences were edited and aligned using SeqMan Ultra version 17.2.0 [15] to derive the consensus sequences for both mtDNA and HSP70 gene fragments. Multiple sequence alignment was performed using Multiple Sequence Comparison by Log Expectation (MUSCLE) version 5 [16]. Haplotypes were determined manually and confirmed using DnaSP version 6 [17], and population diversity indices were calculated using Arlequin version 3.5.2.2 software [18] for both mtDNA and HSP70. Domestic goat mtDNA hypervariable region-1 and HSP70 gene sequences were used as references during alignment.

### 3.2. mtDNA Phylogenetic Relationships

For mtDNA phylogenetic relationships, the twenty-two goat mtDNA reference sequences representing the six goat haplogroups [3] were downloaded from GenBank and used in assigning Galla goats' mtDNA haplotypes into relevant haplogroups. In addition, sequences from other African and non-African countries, wild goats, and the generated haplotype sequences were also included (Table 2). The phylogenetic relationship was determined using the maximum likelihood algorithm implemented in Molecular Evolutionary Genetics Analysis (MEGA) X [19]. Bootstraps of 1000 replications were used.

A median-joining network was drawn to determine the maternal origin and the relationship between the Kenyan Galla goat populations and goats from other world regions using Network version 10.2.0.0.

### 3.3. mtDNA and HSP70 Gene Population Genetic Structure

To determine the genetic differentiation within and among populations, analysis of molecular variance (AMOVA) was performed using Arlequin version 3.5.2.2 [18] for both mtDNA and HSP70 gene haplotypes. In addition, to assess the nonrandom association between genetic differentiation (FST) and geographic distances between populations, a Mantel test was used to plot the regression plot of genetic and geographic distances for both mtDNA and HSP70 gene using GenAlEx v6.501 software [20].

Determination of pairwise fixation index (FST) and inbreeding coefficients (FISs) for the HSP70 gene was also performed using Arlequin version 3.5.2.2.

### 3.4. Demographic Analysis

Demographic history was investigated using the mismatch distribution patterns based on the pairwise differences between mtDNA haplotypes and tests of neutrality. The goodness of fit of the mismatch distribution was confirmed by the sum of the squared deviation (SSD) and Happending's raggedness index (r) using Arlequin version 3.5.2.2 [18].

## 4. Results

### 4.1. mtDNA Genetic Variation and Genetic Diversity

The partial goat mtDNA D-loop region analyzed in this study corresponds to nucleotide positions 15687–16287 of the *Capra hircus* mtDNA reference sequence (GenBank accession no. NC_005044.2). From the 90 consensus sequences generated, 90 polymorphic sites were identified. The polymorphic sites defined 68 haplotypes, where 66 were unique, while two were shared between individuals from Garissa and Tana River counties, indicating similar maternal origin or gene flow between the two counties. Maternal genetic diversity parameters for Galla goat populations are presented in Table 3. All populations showed a high genetic diversity as demonstrated by haplotype diversity ranging from 0.8939 ± 0.0777 in Madogo to 1.0000 ± 0.0221 in Adele goat populations. Nucleotide diversity was the highest in the Raya goat population (0.029578 ± 0.015443) and lowest in the Sankuri goat population (0.013112 ± 0.007365).

### 4.2. Phylogenetic Relationship

**Figure 3:**
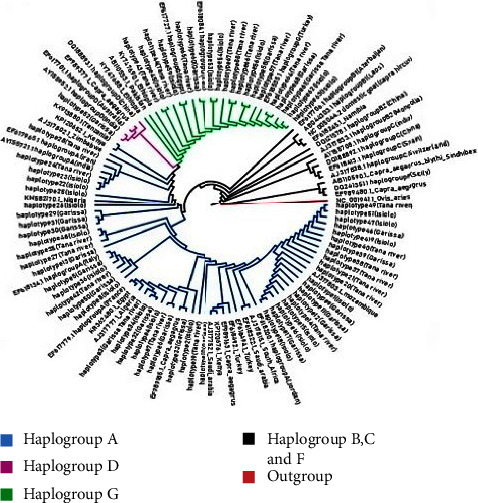
A maximum likelihood tree showing the phylogenetic relationship of Galla goats in Kenya compared to other goat sequences. The model used was Hasegawa–Kishino–Yano (HKY) with a gamma shape parameter of 0.45.

**Figure 4:**
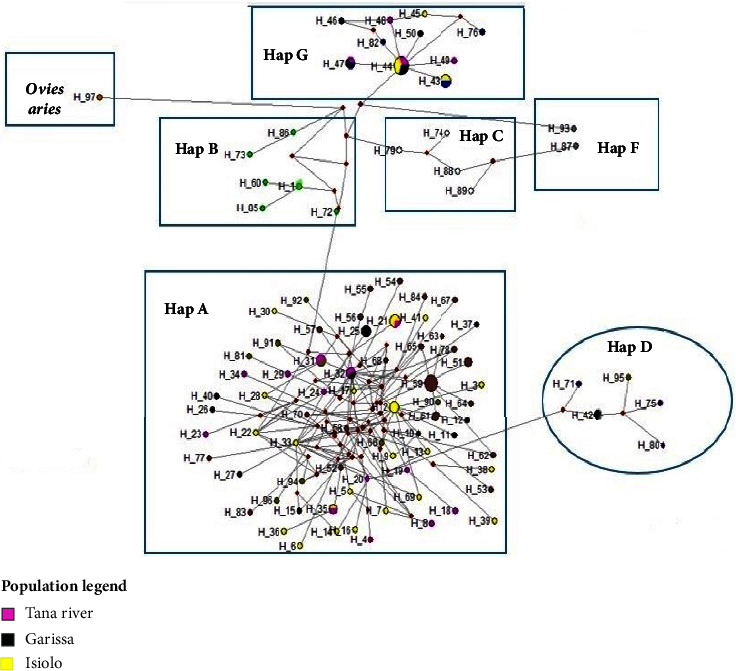
A median-joining network showing the relationships of mtDNA haplotypes from Galla and other worldwide goats.

The phylogenetic relationship was inferred by using a maximum likelihood tree and a median-joining network. Both the tree (Figure 3) and the network (Figure 4) revealed three well-resolved haplogroups in which Galla goats clustered; haplogroups A, D, and G. The three haplogroups could be interpreted as evidence of either three separate maternal origins from genetically distinct populations or one origin from a vast population with three very distinct lineages.

Figure 3 represents a maximum likelihood tree showing the phylogenetic relationship of Galla goats in Kenya compared to other goat sequences. The model used was general time reversible (GTR + G + I) with a gamma shape parameter of 0.3221.

The maximum likelihood tree and median-joining network also showed that haplogroup A was the most predominant consisting of 53 haplotypes representing the majority of Galla goats from the three sampled counties. The distribution of haplotypes in Isiolo, Tana River, and Garissa was 18, 18, and 17, respectively. Most haplotypes from the three counties showed a closer genetic relationship to goat sequences from Ethiopia, Saudi Arabia, and Nigeria than other haplogroup A sequences.

Haplogroup G contained 14 haplotypes from all the counties. Tana River and Isiolo counties had six haplotypes each, while Garissa was represented by four. This haplogroup carries the second largest number of the Galla goat sampled. Galla goat haplotypes in this clade revealed a higher genetic affinity to goat sequences from Iran, Egypt, and Turkey than to other haplogroup G sequences. Haplogroup D comprised only one haplotype (haplotype 52) from Garissa represented by two Galla goats. This haplotype showed a greater affinity to Australian goat sequences than to other sequences in haplogroup D. None of our samples clustered with haplogroups B, C, and F.

From the median-joining network, the geographical patterns of the goat populations were surprisingly weak, as shown by the phylogeographic analysis of the 90 Galla goat sequences. This is because Galla goat samples from Isiolo, Tana River, and Garissa were almost equally distributed in haplogroups A and G. Only haplogroup D had samples from Garissa.

### 4.3. Population Variation Based on mtDNA HVR-1

The analysis based on analysis of molecular variance (AMOVA) indicated that 94.39% of the total genetic variation present in Kenyan Galla goats was explained by genetic differences between individuals within populations and 7.71% (*p*=0.016) among populations within groups. The pairwise AMOVA differences across all goat populations are shown in Table 4.

The high genetic variation within the populations can be explained by mutations in the genetic material and migration into a population. On the other hand, the extremely low genetic variation observed among groups indicates restricted gene flow or a small population size.

**Figure 5:**
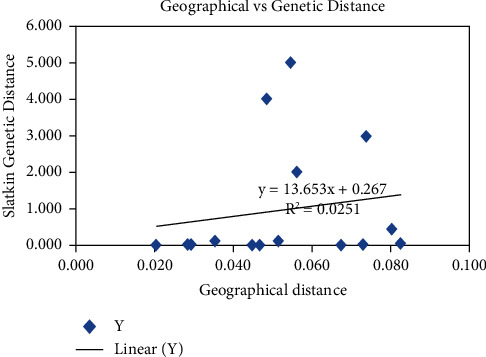
A Mantel regression graph showing the correlation between geographic and mtDNA genetic distance matrices of Galla goats in Kenya.

A regression graph was drawn to determine the correlation between genetic variation and geographical distance (Figure 5).

A weak positive correlation (*r* = 0.158) was found between geographic location and genetic variation in Galla goats in Kenya. The graph indicated a slight increase in genetic variation with increasing geographic distance.

### 4.4. Demographic History

**Figure 6:**
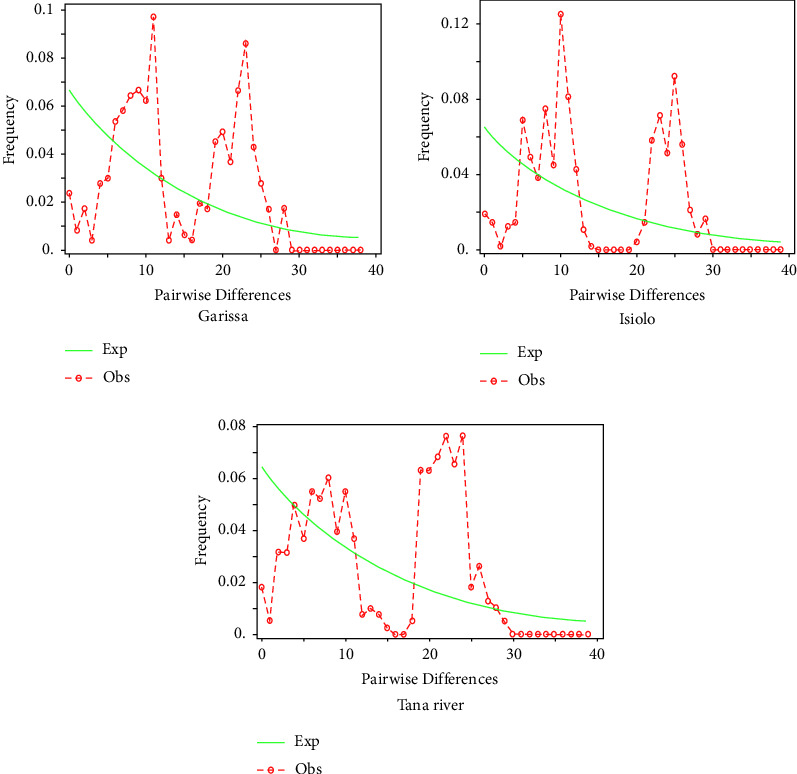
Mismatch distribution patterns. Bimodal mismatch distribution patterns were observed in all the sampled populations.

Past expansion events of Galla goat populations were inferred based on mismatch distribution patterns (Figure 6) and neutrality tests (Table 5). All populations showed bimodal and irregular mismatch patterns indicating population contraction or expansion. Population expansion was further supported by the small but nonsignificant SSD and raggedness index, negative Tajima D, and Fu's Fs neutrality tests in Garissa and Isiolo. In contrast, the Tana River had a positive value which shows population contraction.

### 4.5. HSP70 Gene Diversity

Twenty-one HSP70 haplotypes inferred by six polymorphic sites were observed. The six polymorphic sites were observed at positions 19, 28, 1228, 1445, 1570, and 1658 of the edited HSP70 gene (1757 bp) and consisted of one transition and five transversion mutations. A high haplotype diversity was observed in all the Galla populations as a result of high genetic heterogeneity (Table 6).

The study also reported higher expected (*H*_*E*_) than observed (*H*_*O*_) heterozygosities in all counties, which show deviation of Hardy–Weinberg Deviation.

### 4.6. HSP70 Gene Genetic Structure

Analysis of molecular variance revealed that variation among individuals within populations was the greatest, which can be explained by gene rearrangement. Similar to mtDNA results, among populations variation (13.44%) was the lowest due to limited gene flow between Galla goats in Isiolo, Garissa, and Tana River counties. Also, the low variation within individuals (21.90%) in all populations is associated with the fact that the HSP70 is an important gene for thermoresistance possibly under great selection.

The overall inbreeding coefficient index (FIS), which measures the reduction in heterozygosity, was high at 0.746, a sign of possible nonrandom mating within individuals in all the populations studied. For the population structure, the significant FST showed a moderate differentiation of 0.134. The results of genetic variation inferred using AMOVA are summarized in Table 7.

**Figure 7:**
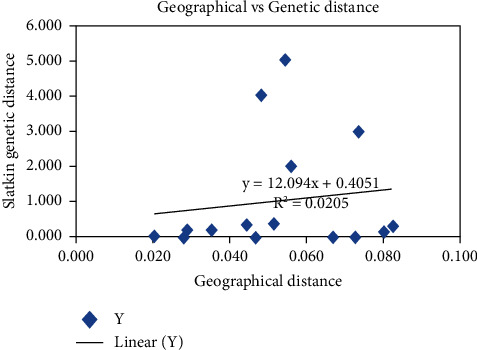
A regression graph showing the relationship between geographic and genetic distance matrices of Galla goat populations in Kenya. The graph was constructed using GenAlEx v6.501 software.

To determine the relationship between genetic variation and geographical distance, a regression graph was drawn (Figure 7). The correlation value (r) revealed a weak positive relationship between genetic variation and geographical distance among the studied counties. This showed that genetic variations increased as the geographical distance increased from Isiolo to Tana River county.

The high inbreeding coefficients (FIS) reported in the three counties showed increased inbreeding rates in all the populations which could be a result of nonrandom mating. All populations had a significant *p* value as shown in Table 8.

## 5. Discussion

Various studies have shown high genetic diversity in African indigenous goat populations [21–24]. With increased temperatures and recurring droughts resulting from climate change, especially in the Horn of Africa, livestock adaptation to the environment is critical in production. Therefore, genetic diversity in indigenous goats would be essential for genetic improvement programs that rely on their resilience and adaptation capabilities to improve production and thus ensure food security. This study evaluated the genetic variation in Galla goat populations from Kenya's arid and semiarid lands. It also gives insights into their genetic origin and demographic history.

### 5.1. MtDNA Genetic Diversity and Phylogenetic Relationship

Our findings revealed a high genetic diversity as portrayed by the high within-population nucleotide and haplotype diversities. Previous studies also detected high haplotype diversities ranging from 0.8 to 1 in Galla goats [25, 26]. The high level of genetic diversity reported could be attributed to the high rate of mitochondrion mutation [27], dispersion from different domestication regions [28], population intermixing, and the probability of multiple wild ancestors [29]. Garissa and Tana River counties shared two haplotypes (3 and 61) which could result from the transhumance of pastoralists in search of water and pasture. This is the most notable characteristic of the pastoral production systems in Kenya. The two counties are separated by River Tana, which serves as a water source, especially during the dry season. As a result, pastoralists travel for a long distance with their animals to the river and then camp for several days. During this period, Galla goats from Garissa and Tana River interact, enabling mating between individuals from the two populations. Mating results in gene transfer leading to possible haplotype sharing between the counties [30]. Haplotype sharing could also have happened due to trade or social gifts such as bride prices between the two counties.

By utilizing the known goat mtDNA-based haplogroup classification system [3], three out of the six haplogroups (A, D, and G) were revealed. The three distinct haplogroups could be interpreted as evidence of either three different maternal ancestries from genetically distinct populations or one ancestry from a vast population with three very divergent lineages [7]. Since such divergent lineages are unlikely to have evolved from a single ancestral population, many studies have challenged the assumption that there is only one maternal origin for goats. Multiple maternal origins are supported by the latest archaeological data consistent with at least two different sites for goat domestication [2]. Mitochondrial DNA studies in other domestic animals, especially pigs, sheep, and cattle, have also revealed several distinct lineages, one of which exists mainly in South or East Asia [31]. This implies a possible Asian origin in addition to other centres in the Fertile Crescent, which supports the hypothesis that multiple maternal ancestries are a common theme in livestock species, including goats [31].

The prevalence of haplogroup A in Kenyan Galla goat populations was expected since it is the oldest [3] and most diverse, and its distribution was similar to the world scenario described in previous studies [32, 33]. Naderi et al. [3] suggested that haplogroup A might have originated from Eastern Anatolia, which is consistent with our study since our sequences clustered with goat sequences from the same region. The results also inform us about a probable second wave of dispersion of goats into Africa through Saudi Arabia and Ethiopia.

Haplogroup G was the second represented among the Galla goats and was previously reported in Kenya by Kibegwa et al. [26]. This corresponded with other studies since haplogroup G is the second most diverse haplogroup worldwide and was first described in East and Northeast Asia [3]. In support of previous studies, our samples showed a higher affinity to Iran, Egypt, and Turkey goat sequences, suggesting similar maternal origin and dispersal routes. This could also indicate the first wave of goats' dispersion from the main domestication centre into Africa.

Haplogroup D was represented by only one haplotype from Garissa, which was more related to Australian goat sequences. Previous studies by Githui et al. [25] and Kibegwa et al. [26] did not report the presence of haplogroup D in Kenya. This haplogroup is mainly found in Northern Europe and Central and Southern Asia [3, 34], and therefore the presence of only one haplotype could be a result of genetic pollution of exotic breeds [35].

The study of ancient DNA has developed from a fringe area that at first attracted little attention to a burgeoning topic that has yielded important insights into the evolutionary paths taken by a number of species [36]. The central domestication area is believed to be around a Fertile Crescent, often called the “cradle of civilization” [2]. Goat dispersion from the centre of domestication could have happened through commercial trade and human migration [2], leading to the present distribution. Turkic languages serve as a common language for a wide range of ethnic groupings that together make up the Turkic peoples. These communities have spread throughout a wide region, encompassing Afghanistan, the Middle East, the Caucasus, Siberia, Northwest China, Central Asia, East Europe, and Anatolia through nomadic migrations [37]. These regions serve as the domestication home for haplogroups A and G [3]. Studies have shown that around 7000 YBP (years before present), goat herds spread into North Africa, especially Egypt and the larger Horn of Africa, either by crossing the Sinai Peninsula or navigating the Mediterranean Sea [7]. This coincided with the opening of a grassland niche in the Sahara that was increasingly occupied by pastoralists, as evidenced by numerous rock paintings of livestock [7]. During the same period, Afro-Asians also spread to the North and Horn of Africa, which could probably mean that they were the livestock owners since many Afro-Asiatic speakers are still pastoralists in that region to date [38]. Afro-Asiatic is a language family of about 300 languages, primarily spoken in the geographic subregions of West Asia, North Africa, the Horn of Africa, and parts of the Sahara/Sahel [39]. The phylum has six branches which are as follows: Berber, Chadic, Cushitic, Egyptian, Semitic, and Omotic, which are native to the African continent except for the Semitic speakers. The Cushitic languages are a branch of the Afro-Asiatic family spoken primarily in the Horn of Africa, with minorities speaking Cushitic languages to the north in Egypt and Sudan and to the south in Kenya and Tanzania [39]. After the domestication of goats and other livestock, which is believed to have majorly taken place in Asia, some Afro-Asians likely left Asia for Africa with their livestock as either traders or pastoralists.

The Phoenicians, Ionians, Romans, and the Arab tribes (Semitic) occupied the Arabian Peninsula before the spread of Islam around 700 CE. This confirms that some of the ancient Semites were pastoralists [40]. After the rise of Islam, the tribes spread to other parts, including North Africa [41], where their African counterparts adopted pastoralism.

**Figure 8:**
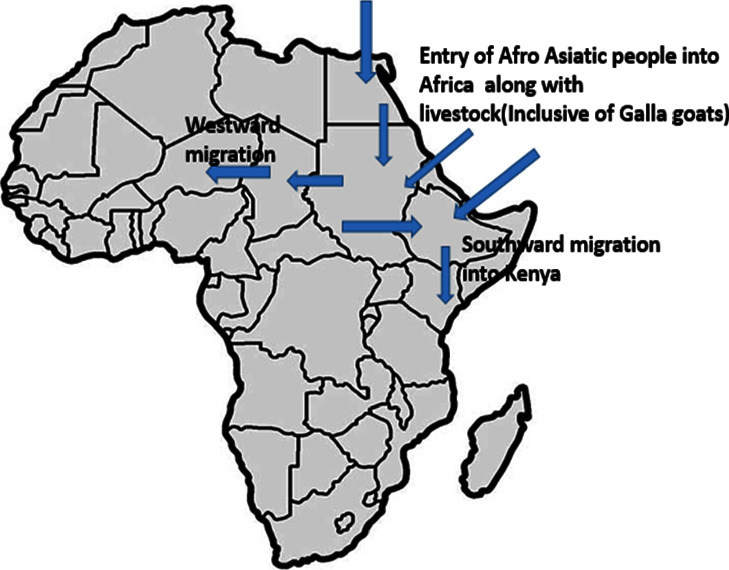
Map of migratory routes of the Galla goat into Kenya. It shows possible dispersion routes of Galla goats into African countries from the centre of domestication.

Climate and environmental changes leading to the desertification of the Sahara and the southward retreat of the tsetse fly belt stimulated the migration of pastoralists to the Sahel region, upper White Nile, and finally into Kenya [7]. The Sahel countries include the following ten African countries: Senegal, Mauritania, Mali, Burkina Faso, Algeria, Niger, Nigeria, Chad, Sudan, and Eritrea. Some of the Cushitic subgroups of the Afro-Asiatic migrated from the Ethiopian highland and the Horn of Africa by 1000 AD. They settled in the Northern Eastern parts of Kenya, where they still practice pastoralism [42]. Historical linguistic and archaeological research suggest that the ancestral southern Cushites migrated to the Turkana region from northern Ethiopia around 5000 years ago and later migrated to Tanzania, where their descendants can be found today [42]. The southward dispersion route of the Galla goats can also be confirmed by the presence of Afro-Asian speakers in Saudi Arabia, Egypt, Tanzania, and Ethiopia, the countries with which Kenyan Galla goats shared haplotypes. Therefore, Afro-Asians likely owned Galla goats since the breed is named after one of the Cushitic subgroups. The map in Figure 8 shows the possible dispersion routes for Galla goats from the centre of domestication.

Haplotype sharing observed between Kenya, Saudi Arabia, Ethiopia, Egypt, and Tanzania is the proof of the north gateway route for haplogroups G and A among Galla goats either independently or in companion. Haplotype sharing suggests the probability of a common maternal origin or goat dispersion pattern found in the wider Horn of Africa region [43]. Like our findings, Gifford-Gonzalez and Hanotte [44] also reported the introduction of domestic goats into the African continent by the Sinai Peninsula, the Mediterranean coast, the Nile Delta, and the Red Sea hills.

The median-joining network also revealed a weak phylogeographic structure among the Galla goats because they did not cluster based on the populations. The absence of phylogeographic structure could be a common feature of domestic goats since it has been reported in other studies by Nguluma et al. [27] and Luikart et al. [31]. The ease of transportation of goats, their use as articles of commerce and sociocultural exchange, and their inherent ability to adapt to a variety of production and ecological environments, e.g., compared to cattle, have been used to explain the lack of phylogeographic structure and high level of genetic diversity (Tarekegn et al.) [43].

### 5.2. mtDNA Genetic Variation and Demographic History

The high genetic variation observed within populations could be attributed to a lack of selective breeding, random mating within the population, mutations that can generate completely new alleles in a population, or even reshuffling of alleles within an organism's offspring during homologous recombination [45, 46].

The observed low genetic variation among the Galla goat groups can be associated with restricted gene flow between the counties, small population, and inbreeding of Galla goats within each county [47]. Gene flow can be defined as the transfer of genes among populations and is facilitated by physical proximity and restricted by physical barriers separating the populations despite the political boundaries. Isiolo county has several hills and plains which could restrict the movement of Galla goats to Garissa and Tana River counties supporting the unique haplotypes reported in Isiolo county.

Demographic dynamics inferred by mismatch distributions and tests of neutrality showed that Galla goats had undergone population expansion. Mismatch distribution patterns were bimodal, indicating that Galla goats' populations were either in equilibrium or expansion was associated with allopatry [26]. Allopatry refers to the secondary contact of populations after a period of isolation followed by an increase in population over time [48]. The presence of haplogroups A, D, and G among Galla goat populations from different dispersion regions supports the concept of population expansion due to allopatry. Tarekegn et al. [43] associated bimodal patterns of mismatch distribution with two independent events of expansion, which could be isolation followed by secondary contact.

The negative Tajima D and Fu's Fs values further confirmed the expansion of the Galla goat populations or positive selection. Positive selection encourages the spread of beneficial alleles in a population, for example, the functional polymorphisms in the HSP70 gene implicated in heat tolerance. Positive selection involves the rise of advantageous mutations of great interest in adaptive or other functional traits [49, 50]. In general, the negative values of Tajima D and Fu's Fs test revealed the presence of scarce mutations in the Galla goat populations, which could result from population growth. The raggedness index and the sum of squared deviation (SSD) values indicated that mismatch and neutrality tests had a proportionate good fit to a model of an expanding population [43].

### 5.3. HSP70 Polymorphisms and Diversity

Six polymorphic sites revealed in the HSP70 gene inferred 21 haplotypes in contrast to studies by Fatima et al. [51] on Sindh ibex from Pakistan, which reported 17 haplotypes. All the populations had a high haplotype diversity which could be attributed to population intermixing, recent expansion as supported by mtDNA results [27]. The polymorphism at position 19 of the edited sequences corresponds to position 74 of the domestic goat HSP70 gene and 22440749 in chromosome 23. This transversion mutation was also reported by Nikbin et al. [11] who associated the SNP with good-quality sperms in Boer goats and linked the quality to the thermoregulatory role of HSP70 in the testes where spermatogenesis occurs. Therefore, the 74 (A > C) SNP could be attributed to thermotolerance in Galla goats.

Analysis of diversity indices revealed more homozygotes than heterozygotes among the Galla goats translating to low genetic variation. This is attributed to inbreeding which leads to restricted gene flow hence gene similarity [52]. Inbreeding was also supported by the lower expected heterozygosity than the observed, which indicates a deviation from the Hardy–Weinberg equilibrium [53]. Hardy–Weinberg equilibrium is a principle that assumes genotype frequencies in a population remain constant from one generation to another when disruptive factors are held constant [54] The principle assumes conditions without mutations, migration, emigration, or selection pressure for or against the genotype and an infinite population [54]. Therefore, in this study, due to the important role of the HSP70 gene in thermotolerance, there is also the possibility of low diversity due to selection pressure of the gene. The possibility of inbreeding among the Galla goats in these populations could be high because of the limited number of bucks. Excess homozygosity could also be attributed to Wahlund's effects which result from population substructure [55]. In this study, the possibility of Wahlunds' effects may be applicable since analysis of molecular variance showed very limited gene flow between Isiolo, Garissa, and Tana River goat populations which are population substructures.

### 5.4. HSP70 Genetic Structure

Analysis of molecular variance revealed that genetic variation was the highest among individuals within populations at 64.66%, which could be linked to intermixing of Galla goat individuals in a population or mutations that create new alleles in a population [46], as revealed by mtDNA results. Variation among populations was the lowest due to the slight possibility of population intermixing, resulting in limited gene flow [56]. The geographical distance between Isiolo, Garissa, and Tana River counties could limit the interaction between the Galla goats hence low genetic variation. These findings concur with the mtDNA results in this study.

Genetic differentiation calculated using the FST index revealed significant moderate differentiation (0.13442). This index is often determined from genetic polymorphism data and ranges from 0 to 1, where a value of 0 means total sharing of genetic material and 1 means no sharing [57, 58]. A fixation index between 0 and 0.05 shows no genetic differentiation among populations, 0.05 to 0.15 signifies a moderate differentiation, while values between 0.15 and 0.25 indicate a high differentiation [59]. The moderate genetic differentiation reported in this study suggests significant gene flow between the Galla goat populations. Like mtDNA, limited gene flow caused by increased distance between populations resulted in isolation by distance (IBD).

Inbreeding coefficients determined using the FIS index revealed a high rate of inbreeding among the Galla goats as revealed by mtDNA results. This index detects the lack or abundance of heterozygotes in each population and the likelihood that two alleles sampled at random are similar by ancestry [60]. The study area practices pastoralism which only favors natural mating where the dominant males exclude weak ones and probably sires most of the offspring [53, 60].

## 6. Conclusions

In this study, the genetic diversity and population dynamics of the Galla goats were covered using the mtDNA D-loop and HSP70 gene. We report high genetic diversity among Galla goats, indicating the possibility of sustainable use for enhancement through breeding and the importance of maintaining diversity by applying appropriate conservation strategies. This study also showed that genetic variation accounted for within populations was the highest for both mtDNA and HSP70 gene, likely due to gene flow, mutations, sexual recombination, and absence of selection programs making the region a hotspot for genetic diversity studies and sources of important alleles for breeding purposes. Studying the genetic basis of locally adapted native populations is critical to developing appropriate breeding strategies and programs to enhance and maintain their genetic diversity. Diversity studies are important in the identification of climate-smart livestock breeds which are able to survive the negative effects of climate change. This is crucial for productivity improvement and for promoting food security. Therefore, the information gained from this study is valuable for the national animal breeding program and conservation purposes.

## Figures and Tables

**Table 1:** Summary of the sampled populations.

County	Villages	Male	Female	Total
Isiolo	Ngare Mara	8	8	16
	Daaba	8	8	16

Garissa	Sankuri	8	8	16
	Raya	8	8	16

Tana River	Madogo	8	8	16
	Adele	8	8	16

Total		48	48	96

**Table 2:** GenBank accession numbers for mtDNA reference sequences.

NC_005044.2_domestic goat	AB110553.1_Pakistan
KP120653.1_Kenya	AJ317752.1_Saudi Arabia
KP120676.1_Kenya	AJ317753.1_Saudi Arabia
KP120652.1_Kenya	EF618242.1_Namibia
KY747690.1_Ethiopia	EF618245.1_Namibia
KY747691.1_Ethiopia	AJ317804.1_Mozambique
KM582170.1_Nigeria	AJ317803.1_Mozambique
AB110552.1_Pakistan	EF618240.1_Mozambique
AJ317812.1_South Africa	EF618241.1_Mozambique
AJ317815.1_South Africa	KJ466263.1_South Africa
EF618351.1_South Africa	AJ317803.1_Zimbabwe
AJ317802.1_Zimbabwe	AJ317777.1_Algeria
EF618545.1_Zimbabwe	KP120679.1_Kenya
AJ317779.1_Algeria	KY747691.1_Ethiopia
KP120678.1_Kenya	KY747692.1_Ethiopia
KP120677.1_Kenya	KY747688.1_Ethiopia
KR362478.1_Egypt	KY747689.1_Ethiopia
KR362479.1_Egypt	EF617706.1_haplogroupB1 (Azerbaijan)
KR362480.1_Egypt	DQ121578.1_haplogroupB2 (China)
EF618322.1_Saudi Arabia	DQ188892.1_haplogroupC (China)
EF617701.1_haplogroupD (Australia)	DQ188893.1_haplogroupD (China)
EF617727.1_haplogroupG (Egypt)	AY155708.1_haplogroupC (India)
EF617779.1_haplogroupA (France)	AY155952.1_haplogroupD (India)
AY155721.1_haplogroupA (India)	EF617965.1_haplogroupA (Iran)
EF618084.1_haplogroupG (Iran)	EF618134.1_haplogroupA (Italy)
EF618200.1_haplogroupA (Jordan)	AB044303.1_haplogroupB1 (Laos)
AJ317833.1_haplogroupB2 (Mongolia)	DQ241351.1_haplogroupF (Sicily)
EF618413.1_haplogroupC (Spain)	EF618535.1_haplogroupG (Turkey)
AJ317838.1_haplogroupC (Switzerland)	EF618492.1_Turkey
EF618494.1_Turkey	KX913779.1_SEA_Gogo (Tanzania)
KX913920.1_SEA_Sonjo (Tanzania)	KX913820.1_SEA_Sukuma (Tanzania)
KX913880.1_SEA_Pare (Tanzania)	EF989480.1_Capra aegagrus
AB110590.1_Capra aegagrus_blythi_Sindh ibex	EF989163.1_Capra aegagrus
EF989377.1_Capra aegagrus	EF989185.1_Capra aegagrus
NC_001941.1_Ovis aries	

**Table 3:** Genetic diversity of Galla goat populations based on mtDNA HVR-1.

	Villages	Sample size	Number of polymorphic sites	Number of haplotypes	Haplotype diversity (Hd ± sd)	Nucleotide diversity (*π* ± sd)
Isiolo	Ngare Mara	14	45	12	0.9670 ± 0.0437	0.024268 ± 0.013020
	Daaba	17	52	13	0.9632 ± 0.0328	0.026965 ± 0.014187

Garissa	Sankuri	13	30	11	0.9744 ± 0.0389	0.013112 ± 0.007365
	Raya	18	57	14	0.9673 ± 0.0298	0.029578 ± 0.015443

Tana river	Madogo	12	33	8	0.8939 ± 0.0777	0.024386 ± 0.013267
	Adele	16	48	16	1.0000 ± 0.0221	0.026956 ± 0.014243

**Table 4:** Population genetic structure of mtDNA haplotypes from AMOVA.

Source of variation	Degrees of freedom (df)	Sum of squares	Variation	Percentage variation	P value
Among groups	2	22.348	−0.15702	−2.10	0.65885 ± 0.01272
Among populations within groups	3	46.704	0.57735	7.71	0.01662 ± 0.00393
Within populations	84	593.747	7.06842	94.39	0.00587 ± 0.00219

*α* = 0.05.

**Table 5:** Population demographic parameters estimated from the analysis of the mtDNA D-loop fragment.

Counties	Sample size	SSD (*p*value)	Raggedness index (r) (*p* value)	Tajima's D (*p* value)	Fu's Fs (*p* value)
Isiolo	31	0.030 (0.070)	0.023 (0.010)	−0.003 (0.549)	−5.176 (0.05)
Garissa	31	0.0186 (0.130)	0.014 (0.180)	−0.522 (0.329)	−3.34847 (0.120)
Tana River	28	0.0217 (0.150)	0.011 (0.400)	0.089 (0.626)	−7.15907 (0.007)

**Table 6:** HSP70 diversity indices.

County	Polymorphic sites	Homozygotes	Heterozygotes	Hd ± sd	*H*_*O*_	*H*_*E*_
Isiolo	6	32	12	0.918 ± 0.010	0.272	0.918
Garissa	5	28	14	0.823 ± 0.029	0.333	0.823
Tana River	6	24	16	0.741 ± 0.044	0.400	0.741

Hd, haplotype diversity; *H*_*O*_, observed heterozygosity; *H*_*E*_, expected heterozygosity.

**Table 7:** Population genetic structure of HSP70 gene from AMOVA and fixation indices.

Source of variation	Degrees of freedom (DF)	Sum of squares	Variation	Percentage variation	P value	Fixation indices
Among populations	2	25.962	0.136	13.44	≤0.01	FST: 0.134
Among individuals within populations	123	188.705	0.655	64.66	≤0.01	FIS: 0.746
Within individuals	125	28.000	0.222	21.90	≤0.01	

*α* = 0.05.

**Table 8:** Galla goat population inbreeding coefficients.

Regions	Population	FIS	P value
Isiolo	Daaba	0.874	≤0.01
	Ngare Mara	0.794	≤0.01

Garissa	Raya	0.749	≤0.01
	Sankuri	0.620	≤0.01

Tana River	Madogo	0.786	≤0.01
	Adele	0.428	≤0.01

*α* = 0.05.

## Data Availability

The sequence data used to support the results of this study have been deposited in the National Centre for Biotechnology Information (NCBI) sequence reading archive under accession numbers GenBank: OR025677–OR025697 for Galla goats heat shock protein 70 haplotypes and GenBank: OR025698–OR025765 for Galla goats mitochondrial DNA haplotypes.
